# Radiotherapeutic Strategies in the Management of Low-Risk Prostate Cancer

**DOI:** 10.1100/tsw.2010.179

**Published:** 2010-09-14

**Authors:** Kevin S. Choe, Stanley L. Liauw

**Affiliations:** ^1^ Department of Radiation Oncology, University of Texas, Southwestern Medical Center, Dallas, TX, USA; ^2^ Department of Radiation and Cellular Oncology, University of Chicago Hospitals, Chicago, IL, USA

**Keywords:** prostate cancer, radiation, radiotherapy, brachytherapy, hypofractionation, stereotactic body radiotherapy

## Abstract

Prostate cancer is the most common nonskin malignancy among men in the United States. Since the introduction of screening with prostate-specific antigen (PSA), most patients are being diagnosed at an early stage with low-risk disease. For men with low-risk prostate cancer, there exists an array of radiotherapeutic strategies that are effective and well tolerated, such as external-beam radiotherapy and brachytherapy. In recent years, there have been tremendous advances in the field of radiation oncology that have transformed the way radiation is used to treat prostate cancer, such as intensity-modulated radiotherapy, image-guided radiotherapy, and stereotactic radiotherapy. It is now feasible to deliver high doses of radiation to the target volume with improved precision and spare more of the neighboring tissues from potentially damaging radiation. Disease outcomes are generally excellent in low-risk prostate cancer. Improvements are expected with further integration of innovative technologies in radiation delivery, tumor imaging, and target localization.

## INTRODUCTION

Prostate cancer is the most common noncutaneous malignancy among men in the U.S., with more than 180,000 new cases diagnosed each year[1]. Due to widespread screening with prostate-specific antigen (PSA), prostate cancer is diagnosed at a much earlier stage than previously possible and the majority of the patients are diagnosed with low-risk disease[2,3]. For these patients, regardless of treatment modality, excellent outcomes can be expected with a very low risk of mortality from the disease[4]. There are three standard treatment approaches for patients with low-risk prostate cancer: active surveillance, surgery, and radiotherapy. For certain patients, including those who are elderly and with significant comorbidities, active surveillance may be preferable; this approach is discussed separately in this issue of the journal. The two main therapy options are radical prostatectomy and radiotherapy. Although data formally comparing the two modalities are scarce, analysis of several large institutional series suggests equally favorable outcomes for low-risk prostate cancer[5,6,7].

Radiation is an important treatment modality for prostate cancer and there exists a diverse array of radiotherapeutic strategies to treat the disease, including conventional external-beam radiotherapy and brachytherapy. There has been substantial progress in the field of radiation oncology in recent years, especially with regards to tumor localization and precise delivery of radiation. Historically, it was challenging to deliver high doses of radiation to the prostate without incurring significant toxicity, since the gland is situated in such close proximity to the bladder and the rectum. However, more recently, the prostate has become a disease site that showcases the potential of new technologies in radiation oncology. Intensity-modulated radiotherapy has allowed the sculpting of radiation doses around complex target volumes and sparing of neighboring organs. Innovations in image-guided radiotherapy have led to accurate localization of the target before each treatment, and target coverage and normal tissue sparing have improved even further. Importantly, the integration of these more advanced technologies has been shown to result in improvements in both disease control and associated toxicity[8,9]. Radiotherapeutic options in the management of low-risk prostate cancer will be reviewed here.

## LOW-RISK PROSTATE CANCER

Prostate cancer is commonly stratified into three risk groups based on the Gleason score (GS), initial PSA, and clinical staging[10]. Low-risk prostate cancer is defined as those patients that have a GS of 6 or less, PSA of 10 ng/ml or lower, and clinical stage T2a or lower. Because low-risk prostate cancers generally pose little threat to survival, especially in elderly patients with significant comorbidities, identification of men who will most likely benefit from curative therapy is of great interest. If the small subset of patients within the low-risk group who will experience rapid disease progression could be predicted, a large number of patients could be spared from unnecessary treatment-related morbidity and the consequent cost savings would be substantial. Several other potential prognostic factors have been studied, such as PSA doubling time[11], presence of perineural invasion[12,13], pretreatment PSA velocity[14], and percentage of positive biopsy cores[15,16,17]. Consideration of these factors may allow further refinement of risk.

Imaging of prostate cancer may also enhance risk stratification by identifying adverse features that are difficult to appreciate with digital rectal examination (e.g., extracapsular extension, seminal vesical invasion, lymph node metastasis). Various imaging modalities are utilized for prostate cancer, such as ultrasound, computed tomography (CT), and magnetic resonance imaging (MRI), but among them, MRI is generally considered to be the most useful[18]. Previously, MRI for prostate cancer was mostly used to assess extension of the cancer beyond the prostate, as it was not possible to visualize the cancer within the gland. With the recent advances in the field of magnetic resonance, MRI can now potentially discern tumors from normal glandular tissues, and this capability may provide important information for prognosis and treatment of prostate cancer[19,20].

Another approach to better classify patients and improve patient selection for curative therapy may be to utilize molecular profiling. A number of important molecular pathways of prostate cancer have been identified[21] and several studies have attempted expression profiling of prostate cancer using microarrays for messenger-RNA (mRNA) and micro-RNA (miRNA)[22,23,24]. Although preliminary results are very promising, the clinical utility of such expression-based biomarkers remains to be determined[25]. As our understanding of prostate cancer biology deepens, our ability to classify prostate cancer reliably will certainly improve.

## RADIOTHERAPEUTIC OPTIONS FOR LOW-RISK PROSTATE CANCER

As mentioned previously, there is a myriad of treatment options for low-risk prostate cancer that utilize radiation therapy, such as conventional external-beam radiotherapy, stereotactic body radiotherapy, and brachytherapy. It is unclear and controversial which of these modalities offers the most favorable risk/benefit ratio. Randomized trials comparing them do not exist, but fortunately, cure rates are high across all modalities[7,26]. With regards to hormonal therapy, although there is an established role of combining hormonal therapy with radiation therapy in the treatment of intermediate- or high-risk prostate cancer[27,28,29,30], there is no such role in the treatment of low-risk disease. The optimal radiotherapeutic approach is generally determined individually after careful consideration of patient preference, morbidity profiles, and availability of expertise.

Another important consideration when comparing different treatment modalities for prostate cancer is the definition of treatment failure. In the PSA era, a rise in serum PSA is commonly used as the first sign of treatment failure and this is termed biochemical failure. Unlike radical prostatectomy, after which PSA is expected to become undetectable, after radiation, since some normal glandular tissues remain, PSA values decline gradually over several years and may reach a nadir that is still detectable. A consensus panel put forth a recommendation in 2005 on the classification of biochemical failure after radiotherapy, which is now widely used. The so-called Phoenix definition defines biochemical failure after radiotherapy as a PSA rise of 2 ng/ml or more above the post-treatment nadir[31].

## EXTERNAL-BEAM RADIOTHERAPY (EBRT)

EBRT entails daily delivery of radiation to a target volume over a course of several weeks (typically 7–9 weeks). This approach is the most extensively used and tested radiation modality for prostate cancer. Its efficacy appears to be comparable to that of radical prostatectomy, although it results in different toxicities[32,33]. With the modern techniques of delivering radiation, outcomes are excellent for patients with low-risk disease. For example, in a study that used intensity-modulated radiotherapy to a dose of 86.4 Gy, the 5-year PSA relapse-free survival for low-risk patients was 98%[34].

### Conventional Radiotherapy

Highly penetrating megavoltage radiation beams from cobalt machines and high-energy linear accelerators have allowed the delivery of tumoricidal doses to the target volume, while minimizing the damage to the skin and other adjacent organs. Historically, the treatment field for prostate cancer was designed based on plain films and bony landmarks, and the beam arrangement was typically a four-field approach resulting in a radiation volume known as a “four field box”. Due to the difficulty of localizing the tumor or the prostate gland, a large volume was targeted to ensure proper coverage. Consequently, more of the surrounding tissues were included in the target volume and the dose that could be safely delivered was limited to 60–65 Gy. Later, when reconstruction with CT imaging was analyzed retrospectively, it was shown that even with the large safety margin, the tumor was often missed with the conventional technique[35]. It is important to note that many of the long-term outcome data for radiotherapy in prostate cancer are derived from patients treated with the conventional technique and, not surprisingly, outcomes were relatively poor, even in patients with early-stage diseases[36,37].

### Three-Dimensional Conformal Radiotherapy

With the availability of CT-based radiotherapy simulation by the mid-1980s, it became possible to utilize more conformal treatment fields that are shaped to match the prostate target volume. Furthermore, the beam angles are adjusted to maximize target coverage and minimize high-dose exposure to nearby normal organs. With “3-dimensional conformal radiotherapy” (3D-CRT), radiotherapy doses can be escalated without incurring more toxicity (e.g., proctitis and cystitis). In a randomized trial from the Royal Marsden Hospital, conventional radiotherapy was compared to conformal radiotherapy for prostate cancer[8]. Among 225 men who were enrolled in the study, radiation-induced proctitis and bleeding was significantly reduced with conformal radiotherapy compared to conventional radiotherapy (5 vs. 15% Grade 2+ toxicity, $p = 0.01$).

## Dose Escalation for Low-Risk Prostate Cancer

Several randomized trials have shown that a higher radiation dose is associated with improved biochemical outcomes in localized prostate cancer, as summarized in Table 1[38,39,40,41]. However, the subgroup of patients for whom dose escalation is most beneficial has not been clearly identified. It is debatable whether higher doses are necessary in low-risk prostate cancer. For example, in the Dutch randomized trial that compared 68 with 78 Gy, the benefit of higher radiation dose was limited to only intermediate- and high-risk patients and not low-risk patients[42]. The initial report of the MD Anderson randomized study comparing 70 vs. 78 Gy (prescribed to the a point within the prostate called the “isocenter,” rather than the entire prostate volume) also showed that the benefit of dose escalation was mainly seen in patients with PSA greater than 10 ng/ml[43]. However, because the natural history of low-risk prostate cancer is long, longer follow-up may be necessary to see the benefit of dose escalation in this risk group. In fact, with longer follow-up (8.7 years), the MD Anderson trial eventually demonstrated a benefit to dose escalation even in patients with low-risk disease[40]. In addition, a meta-analysis that included seven randomized trials and over 2,800 patients showed that the biochemical failure was significantly reduced with a higher dose of radiotherapy in all risk groups, including the low-risk group[44]. While there is solid evidence that higher doses are associated with improved biochemical outcomes, there have been no differences in clinical end points, such as cancer-specific survival or overall survival. Further maturation of data may be necessary.

**TABLE 1**
**Randomized Trials Investigating Dose Escalation of Radiotherapy**

| Study (Years Open) | Radiotherapy Doses Compared | Outcome | Low-Risk Cohort |
|---|---|---|---|
| MDACC (1993–1998)[40] | 70 vs. 78 Gy | 59 vs. 78% (8-year FFF) | 63 vs. 88% (8-year FFF) |
| MGH-Loma Linda (1996–1999)[41] | 70.2 vs. 79.2 GyE | 68 vs. 83% (10-year FFF) | 72 vs. 93% (10-year FFF) |
| Dutch CKVO96-10 (1997–2003)[38] | 68 vs. 78 Gy | 45 vs. 56% (7-year FFF) | N/A |
| MRC RT01 (1998–2002)[39] | 64 vs. 74 Gy | 60 vs. 71% (5-year bPFS) | 79 vs. 85% (5-year bPFS) |

Abbreviations: MDACC = MD Anderson Cancer Center; MGH = Massachusetts General Hospital; MRC = Medical Research Council; Gy = Gray; GyE = Gray equivalent; FFF = freedom from failure; bPFS = biochemical progression-free survival.

Although dose-escalation can lead to improved biochemical control in prostate cancer, higher doses can also lead to increased toxicity. The MD Anderson trial showed that the risk of Grade 2+ gastrointestinal (GI) toxicity was twice as high in the higher-dose arm (26 vs. 13%)[40]. Strikingly, of the 11 patients who developed Grade 3 proctitis, 10 were in the higher-dose arm[45]. A similar increase in toxicity was observed in other randomized trials as well as the meta-analysis[39,41,44]. For urinary toxicity, none of the trials found significant correlation between the late urinary toxicity and radiation dose. It is possible that urinary toxicity takes longer to manifest and with longer follow-up, a correlation may be revealed[45]. To improve the therapeutic benefit of EBRT for low-risk prostate cancer, an optimal radiotherapy dose that can eradicate the disease without the high risk of toxicity needs to be defined. Advances in radiation delivery techniques will also improve the therapeutic ratio.

## Intensity-Modulated Radiotherapy (IMRT)

One strategy to improve tumor target coverage while limiting high-dose exposure of normal tissues is IMRT. IMRT is a form of 3D-CRT in which the physician defines target volumes, beam angles, and normal tissue tolerances, and a complex computer algorithm derives the optimal treatment plan by dynamically controlling the intensity of the radiation beam[46]. This allows for a high degree of dose conformity around complex and irregularly shaped tumor volumes. An example dose distribution of an IMRT plan to treat the prostate and proximal seminal vesicles is shown in Fig. 1.

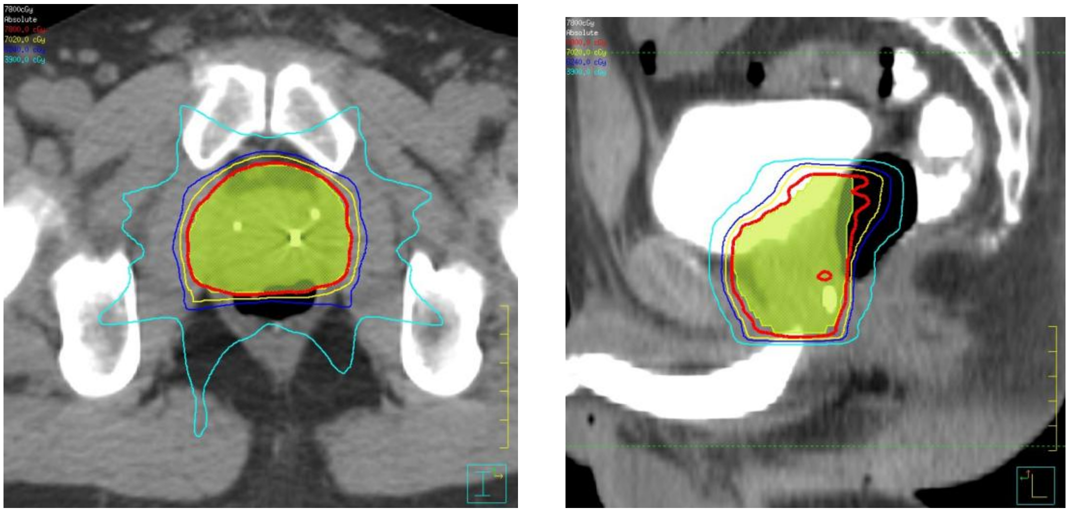

**FIGURE 1.** Axial and sagittal views, representing the 100% (red), 90% (yellow), 80% (blue), and 50% (aqua) isodose lines of a man treated with IMRT to the prostate to a total dose of 78 Gy. The initial planning target volume, consisting of the prostate and proximal seminal vesicles plus a 5- to 8-mm margin, is shaded yellow.

Cumulating evidence suggests that IMRT can improve acute and late toxicities when used for prostate cancer and thus allows safe dose escalation. For example, a study from the Memorial Sloan-Kettering Cancer Center reported their experience of using IMRT in 772 prostate cancer patients to a dose up to 86.4 Gy[47]. The toxicity profiles after IMRT were favorable. The 3-year risk of Grade 2+ proctitis was 4% with IMRT compared to 14% with 3D-CRT.

Although IMRT appears to enable dose escalation with less toxicity, there are a few concerns with IMRT that need to be considered. First, target motion is a particularly important issue when using IMRT. Unlike 3D-CRT, in IMRT, the dose from each beam is not delivered all at once. At each beam angle, the intensity of the beam is “modulated” by multiple smaller subfields that change with time. Due to movement of the prostate related to variation in rectal and bladder filling, the longer time of treatment, and the use of increasingly precise therapy, IMRT has the potential to result in a suboptimal dose distribution (and geographic miss) of the intended target. Another issue in prostate IMRT is the amount of normal tissue exposed to radiation. IMRT may reduce the volume of normal tissues receiving high doses of radiation, but in turn, it substantially increases the volume receiving lower, yet still potentially damaging, doses. The spread of lower-dose radiation to a large area theoretically can increase the risk of radiation-induced malignancy (see below). Finally, IMRT is labor intensive and costly. Analysis of clinical benefit with respect to the cost is necessary and with such information, its appropriate use will be better defined.

## Target Localization and Organ Motion Tracking

The prostate gland is a mobile organ, and it is well documented that position of the prostate can vary substantially with regards to bony landmarks due to factors such as breathing and distension of the rectum and the bladder[48,49,50]. Highly conformal radiation delivery requires precise localization of the prostate so that the radiation can be targeted appropriately. A variety of strategies have been developed to account for prostate motion, including transabdominal ultrasound-based imaging, on-line CT, and implanted radiopaque markers[51,52,53,54,55,56]. For example, radiopaque fiducial markers can be implanted within the prostate gland, which then are visualized on portal imaging and targeting is adjusted accordingly prior to each treatment[51,55]. Because the prostate gland itself is not visualized, this approach does not account for changes in the shape or the size of the prostate gland that may occur during therapy[57]. However, these changes are generally small, and other studies have shown that fiducial markers provide consistent and reliable positioning of the prostate for radiotherapy[58].

The above-mentioned techniques are largely used to track interfractional prostate movement (i.e., motion between treatments). However, they do not account for prostate movement during treatment (intrafractional movement). There is a growing interest in real-time tracking of the prostate. One technology that offers real-time tracking is the Calypso localization system (Calypso Medical Technologies, Seattle, WA). In this system, radiofrequency transponders are implanted in the prostate and tracked in real time, and if the target volume deviates beyond predetermined boundaries, the radiation delivery can be stopped[59]. This approach ensures that the target is treated with radiation appropriately.

Advances in prostate localization techniques can theoretically improve disease control by assuring target coverage and reduce complications by minimizing the volume of normal tissues receiving high doses of radiation. The clinical utility of the new technologies is under active investigation.

## Acute and Late Toxicities Associated with EBRT

Although all cancer therapy is associated with potential toxicity, EBRT for prostate cancer is generally well tolerated and most men continue their usual daily activities throughout the course of therapy. Toxicities from radiation can be categorized into those that occur during therapy (acute toxicity) and those that occur months to years after therapy (late toxicity). Although acute toxicities may be vexing, they generally resolve after the completion of therapy and most patients recover with only supportive measures. On the other hand, late toxicities are more unpredictable and potentially irreversible, and therefore more concerning.

Acute toxicities associated with prostate irradiation are mostly due to the toxic injuries in the bladder and the rectum. Common GI symptoms are frequent loose stools, tenesmus, and cramps. Symptoms related to genitourinary (GU) toxicities may be frequency, dysuria, and urgency. These side effects are common and seen in about half of all treated patients[41,42,60], but they are usually managed with simple antidiarrheal or anti-inflammatory medications, and typically resolve within a few weeks of the therapy completion.

Significant late complications are relatively rare; the risk of moderate to severe late toxicity is about 5–10%[33,38,61]. Late side effects can include rectal urgency, hematochezia, hematuria, and may rarely include strictures, perforation, and incontinence. It has been suggested that patients on anticoagulation therapy may face a particularly high risk of rectal bleeding when treated with EBRT[62] and those who had previous transurethral resection of the prostate (TURP) may have a higher incidence of severe GU toxicity[63]. Use of more modern radiation techniques may mitigate some of the risk for significant complications[47].

Another important sequela of prostate radiotherapy is sexual dysfunction, especially in those who receive hormonal therapy. Properly assessing the impact of radiotherapy on erectile dysfunction is challenging, since most patients with prostate cancer are elderly men who already experience the normal age-related decline in sexual function. In addition, they often have comorbid diseases (e.g., cardiovascular diseases and diabetes) and may take medications that contribute to erectile dysfunction. Nevertheless, decline in erectile function is seen about 30–50% of men after EBRT and the incidence rises with time[64,65,66]. Phosphodiesterase inhibitors can be effective in ameliorating the radiation-induced erectile dysfunction[67,68].

Finally, one potential complication that is often discussed with prostate radiotherapy is the risk of second malignancy. Patients with prostate cancer are generally older and some (5–8%) may develop another malignancy after receiving therapy for prostate cancer[69,70]. Approximately 80% of second malignancies that are diagnosed after prostate cancer treatment are located outside of the pelvis[71]. Several studies have suggested that the risk of developing a second malignancy is higher after radiotherapy compared to surgery or no prior therapy[69,72,73,74]. However, there are also studies reporting no increased risk of second malignancy after radiotherapy[70,75,76]. It is possible that the incidence of second malignancy after prostate cancer treatment may be influenced by other factors, such as more vigilant screening with follow-up. Radiotherapy for prostate cancer may be associated with an increased risk, but the magnitude of such an increase is very small, making its clinical significance unclear[77].

## PROSTATE BRACHYTHERAPY

Brachytherapy is another well-accepted and effective treatment option for low-risk prostate cancer. It involves transperineal implantation of radioactive sources directly inside the prostate. Because of the rapid fall-off of radiation dose beyond the implanted volume, this technique results in high doses within the prostate and immediate vicinity with minimal exposure to the surrounding normal tissues. Permanent seed implant or low-dose-rate (LDR) brachytherapy has been the traditional approach to treat prostate cancer, but more recently, high-dose-rate (HDR) brachytherapy that delivers radioactive sources via an afterloader is also being utilized.

### Low-Dose-Rate Brachytherapy

LDR brachytherapy places metal seeds containing radioactive isotopes into the prostate, using real-time imaging and guidance systems[78]. A variety of isotopes are utilized for LDR brachytherapy that differ slightly in their physical properties (Table 2). Iodine-125 and palladium-103 are the most commonly used isotopes in prostate brachytherapy. The use of cesium-131 has recently gained interest because of its shorter half-life, but experience is much more limited with this isotope. The radioactive seeds are placed predominantly in the periphery of the prostate to target the area of disease and spare the urethra. Radiation from the isotopes is then deposited within the prostate gland as the isotopes decay. The sharp dose fall-off allows for a higher dose that can be delivered, and typical prescribed doses are 145 and 120 Gy for iodine-125 and palladium-103, respectively[79]. An example dose distribution of a brachytherapy plan is shown in Fig. 2.

Brachytherapy planning has traditionally been performed prior to the implant (preplanning). Using a transrectal ultrasound, pictures of the prostate are taken in sequential axial slices. Thereafter, using a computer planning system, the prostate contours are then delineated on these slices. A plan is derived to determine the location and number of needles that need to be inserted, and the number of seeds to be implanted within each needle, to cover the target volume adequately. Because the position of the prostate gland at the time of the planning procedure may not perfectly replicated on the day of the implant, a technique that would allow real-time or intraoperative planning has also been developed. Real-time planning may lead to improved dosimetry and perhaps clinical outcomes. Early reports utilizing real-time planning suggest that conformality and toxicity profiles may be superior[80].

**TABLE 2**
**Comparison of Radioactive Seeds in Brachytherapy**

| Isotope | Half-Life | Energy | 90% Dose | Total Dose |
|---|---|---|---|---|
| Cs-131 | 9.7 days | 30.4 keV | 33 days | 115 Gy |
| Pd-103 | 17 days | 20.8 keV | 58 days | 120 Gy |
| I-125 | 60 days | 28.5 keV | 204 days | 145 Gy |

keV = kiloelectron volt.

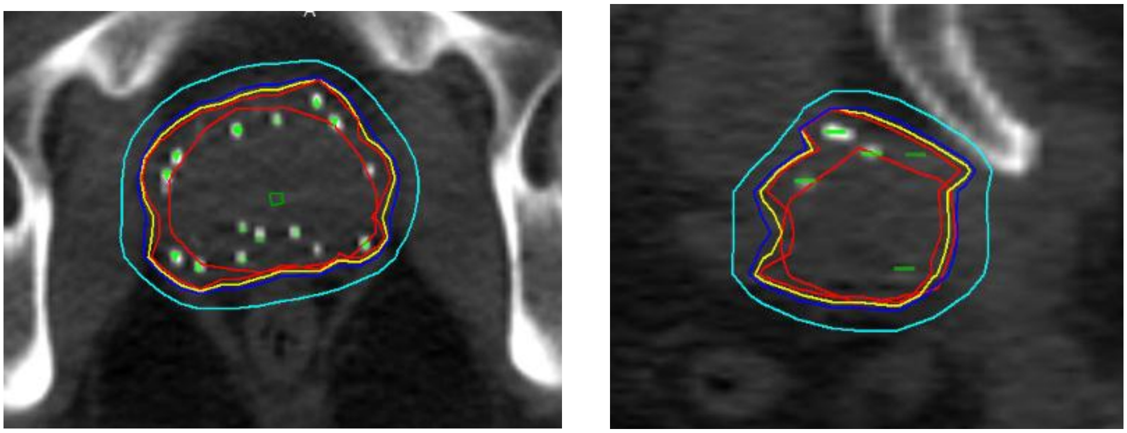

**FIGURE 2**. Axial and sagittal views, representing the 100% (red), 90% (yellow), 80% (blue), and 50% (aqua) isodose lines of a man treated with brachytherapy to the prostate to a total dose of 144 Gy. The clinical target volume, consisting of the prostate, is outlined in red.

## High-Dose-Rate Brachytherapy

HDR brachytherapy or temporary prostate implant entails inserting hollow catheters transperineally into the prostate gland, in a manner similar to inserting needles for LDR brachytherapy. The catheters stay in place for the duration of the implant and a single high-activity source (usually iridium-192) is then placed at specified positions via the catheters for a predetermined duration. Because of the high activity of the source, each dose is delivered in minutes, after which the source is retracted into the afterloader.

Unlike LDR brachytherapy in which the placement of each seed is critical to realize the intended dose distribution, in HDR, dose distribution can be modified after the catheter placement by altering the dwelling time at each position. In addition, in LDR brachytherapy, the radioactive seeds may migrate within the prostate or even to distal sites, but this potential complication is entirely avoided in HDR brachytherapy. Another potential advantage of HDR brachytherapy is that, should the $\alpha/\beta$ ratio be low in prostate cancer as suggested (see below), there may be a radiobiological advantage over LDR brachytherapy[81]. Early reports of using HDR brachytherapy for low-risk prostate cancer are encouraging, with the 5-year biochemical control rates greater than 90%[82,83]. Further follow-up is necessary in order to validate the long-term efficacy of HDR brachytherapy.

## Patient Selection for Brachytherapy

Compared to EBRT, patient selection is more critical for a successful outcome with brachytherapy. According to the American Brachytherapy Society, patients with low-risk disease are appropriate candidates for this treatment approach[79]. Other considerations include the size of the prostate gland, ability to tolerate the operative procedure, and the baseline urinary function. For example, a large prostate volume is associated with increased urinary toxicity, particularly acute urinary retention[79,84], and a volume greater than 60 ml is a relative contraindication for brachytherapy[79]. For patients with a large prostate gland, a short course of neoadjuvant hormonal therapy may be administered to reduce the size[85]. Other relative contraindications include large median lobes, previous TURP, multiple previous pelvic surgeries, significant urinary symptoms, and severe diabetes that may compromise healing[79].

## Clinical Outcomes and Toxicity

As with all other modalities, clinical outcomes after prostate brachytherapy have improved significantly in recent years[86]. Advances in implantation techniques and refinement of patient selection likely contributed to the improvement. In a cooperative group study from the Radiation Therapy Oncology Group (RTOG), investigating iodine-125 brachytherapy in 94 patients with low-risk disease, the 5-year biochemical control rate was 99%[87]. Long-term biochemical control rates are also now becoming available. In a large series with 7 years of median follow-up, the 12-year biochemical control rate for low-risk patients was 88%[88]. In another large multi-institutional series, analyzing 2,693 patients, those with low-risk disease had an 8-year PSA-relapse free survival of 74%[86].

The primary toxicity from prostate brachytherapy is related to the urinary tract and the bladder, although rectal toxicity and sexual dysfunction are also potential complications. Irritative or obstructive urinary symptoms occur in most patients following the implant, but these symptoms generally dissipate by 12 months[89]. Acute urinary retention is a potential side effect that may require an urgent intervention. Fortunately, this is a relatively rare event and according to a recent series of 655 consecutive patients treated with iodine-125 brachytherapy, only 3% developed urinary retention necessitating catheterization[90].

## Comparisons with EBRT

The relative efficacy of prostate brachytherapy compared to EBRT is debatable. However, new data are emerging that show that higher doses delivered by brachytherapy may lead to more complete ablation of the prostate gland and lower PSA nadirs[91]. Since the PSA nadir after radiation is predictive of prostate cancer–specific mortality in patients treated with radiotherapy[92,93], a lower PSA nadir achieved with brachytherapy may translate into improved disease outcomes. Another potential advantage of brachytherapy is the more localized dose distribution and better sparing of the surrounding structures. Whereas in EBRT radiation beams traverse normal organs to reach the prostate, with brachytherapy, the radiation emanates from within and the dose fall-off is sharp beyond the intended target. Consequently, a highly conformal dose distribution is feasible and may lead to improved toxicity, especially for the rectal and sexual function[94,95]. Furthermore, since the radioactive seeds are implanted within the prostate and move with the gland, organ motion is not as much of a concern as it is in EBRT. Finally, LDR brachytherapy is generally an outpatient procedure that is easily tolerated and the recovery period is relatively short. Compared to a 7- to 9-week course of daily EBRT, brachytherapy requires substantially less time commitment and the convenience of this approach may be appealing to patients for whom daily traveling is difficult.

There are potential disadvantages of brachytherapy compared to EBRT. One major drawback is the higher level of uncertainty with regards to achieving the intended dose distribution. The success of the procedure is dependent on the placement of the seeds, and some level of experience and expertise is required to perform a high-quality implant. Another difficulty with brachytherapy may be that although the procedure is generally simple and well tolerated, it is more invasive than EBRT and a small risk of anesthetic complications exists. In addition, perhaps related to the higher biologically effective doses delivered to the prostate gland, there is greater potential for severe urinary morbidity compared to an external beam approach[96]. Finally, because of the sharp dose gradient of the radioactive seeds, brachytherapy has limited capacity to target areas beyond the prostate gland. This constraint limits the utility of brachytherapy as monotherapy in intermediate- or high-risk prostate cancer, and underscores the importance of proper patient selection.

## EMERGING RADIATION TECHNOLOGIES

### Hypofractionated Radiotherapy

As mentioned briefly above, the biological effect of ionizing radiation on target tissues can be characterized by a theoretical quantity called the α/β ratio. For prostate cancer, the α/β ratio is proposed to be much lower than other tumor types[97,98,99], which suggests that prostate cancer cells may be more susceptible to cell killing by larger fraction sizes (i.e., hypofractionation). Hypofractionated radiotherapy would have an added benefit of a shortened duration of therapy, since larger doses are given per day and the cumulative dose is adjusted to a lower dose.

A number of randomized trials have compared hypofractionated radiotherapy with conventional radiotherapy for prostate cancer[100,101,102]. However, many of the trials have been criticized for short follow-up periods and use of radiation doses that are too low by current standards. The Cleveland Clinic reported their hypofractionation experience (2.5 Gy per fraction to 70 Gy) and showed that the 5-year biochemical control for low-risk patients was 94%[26]. This regimen has been compared with standard fractionation (1.8 Gy per fraction to 73.8 Gy) in an RTOG trial and the results are maturing.

An extreme form of hypofractionation or so-called stereotactic body radiotherapy (SBRT) is to deliver very large doses in just a few fractions, using a technique that can deliver radiation in a highly precise manner. A prospective study from Stanford University used 36.25 Gy over five fractions (7.25 Gy per fraction) to treat 41 patients with low-risk disease[103]. No biochemical failures have been observed yet with a short median follow-up of 33 months.

Although the convenience of a shorter treatment course and the theoretical advantage in radiobiology are attractive, noncancerous tissue tends to demonstrate a higher rate of late toxicity with larger doses per fraction. Longer follow-up and further validation are therefore critical before hypofractionated radiotherapy can be adopted as a standard treatment option for low-risk prostate cancer.

### Protons and Other Heavy Particles

Another possible radiotherapy approach to enhance the therapeutic ratio for prostate cancer is to utilize charged particles, such as protons and carbon ions. Dose deposition from standard photon beams is relatively constant over a range in tissue, and some dose to the normal structures proximal and distal to the target is inevitable. On the other hand, charged particles deposit most of their energy at a given depth followed by a steep dose fall-off, with almost no dose deposition beyond what is called a Bragg peak. This physical property can be exploited to deliver higher doses to the target without exposing surrounding normal tissues to potentially damaging doses of radiation.

The clinical benefit of heavy-particle therapy for prostate cancer is unclear. For proton therapy, a dosimetric analysis showed that proton therapy does not offer significant improvement in conformity or sparing of normal tissues over photon IMRT[104]. Data regarding clinical outcomes are also similar to those with standard photon therapy. In a large series of 1,255 patients treated at Loma Linda University, the 5-year biochemical disease-free survival was 73% after proton therapy[105]. The rate of serious toxicity was low and comparable to standard photon therapy and IMRT.

Carbon ion radiotherapy (CIRT) is another new radiation modality using heavy particles. Similar to proton beams, carbon ion beams also produce a Bragg peak and abruptly stop as energy is lost. However, unlike protons, whose biologic properties do not differ much from photons, carbon ions have relative biological effectiveness (RBE) that are up to four times higher than photons and protons[106]. Because of its favorable physical and biological characteristics, CIRT may improve tumor control without causing more toxicity. The effectiveness of CIRT for prostate cancer has been studied in a limited manner. In a prospective trial from Japan with 175 men, the 4-year biochemical control rate was 87% in low-risk patients treated with 66 GyE (gray equivalent) of CIRT[107].

Favorable physical and biological properties of charged particles make them an attractive alternative to conventional photon radiotherapy. However, these technologies are still in their infancy, and compared to 3D-CRT and IMRT, planning and delivery of charged particles are relatively primitive. In addition, the cost of heavy-particle therapy in construction and operation may be prohibitively high. With more experience and reduced cost, heavy-particle radiotherapy may become an integral component of prostate cancer therapy.

## RECOMMENDATIONS

Controversies and uncertainties abound when deciding the best treatment for low-risk prostate cancer. Because the expected outcomes from the cancer are excellent, perhaps even with no therapy, it is essential to be mindful of the potential toxicities associated with treatment. Although the disease outcomes are generally equivalent among major treatment options, the toxicity profiles can be very different[33]. When deciding which treatment modality is optimal, many factors are considered, including life expectancy, disease extent, prostate size, and patient preference. Advantages and disadvantages of each treatment option should be discussed thoroughly so that an informed decision can be made.

Most men with low-risk prostate cancer who require treatment will be good candidates for a radiotherapeutic approach. EBRT is a noninvasive treatment option that can be safely offered to almost all patients. For patients who are at higher risk for anesthetic complications, EBRT is typically the preferred treatment modality. It may also be preferred for patients with significant pre-existing urinary obstructive symptoms, history of TURP, or large prostate glands, since brachytherapy is associated with a higher risk of toxicity in these patients. In patients who have a higher risk of radiation-related toxicity or shorter life expectancy, the EBRT dose may be tailored in consideration of the individual’s risk/benefit ratio.

For patients who are relatively young with a longer life expectancy (>15 years) and no contraindications otherwise, prostate brachytherapy (either LDR or HDR) may be the best option. Although the outcomes after brachytherapy and EBRT have not been directly compared in a randomized setting, men treated with a high-quality brachytherapy procedure for low-risk prostate cancer are more likely to obtain a lower post-treatment PSA nadir than men treated with EBRT[91,108]. It is reasonable to infer that the lower PSA nadir can translate into more favorable long-term disease control, knowing that some late recurrences result from incompletely treated local disease[109]. Prostate brachytherapy is usually tolerated well with favorable toxicity profiles, especially with regards to rectal and sexual toxicities, and the treatment course is much shorter than EBRT.

For select patients who prefer a noninvasive treatment that can be completed within a short period of time, hypofractionated radiotherapy or stereotactic body radiotherapy may be considered. However, caution is warranted with this approach, since long-term outcomes are not yet available. It is recommended that utilization of this approach should be within the context of investigational protocols.

Recent advances in the planning and delivery of radiotherapy have transformed the radiotherapeutic landscape for low-risk prostate cancer. Many strategies have been developed that are more effective and better tolerated than conventional radiotherapy. New technologies, such as IMRT, have enabled dose escalation and improved sparing of the adjacent normal tissues. Further improvement in the therapeutic ratio is anticipated with the utilization of innovative tumor imaging and target localization.
